# Fine-scale genetic mapping of a hybrid sterility factor between *Drosophila simulans *and *D. mauritiana*: the varied and elusive functions of "speciation genes"

**DOI:** 10.1186/1471-2148-10-385

**Published:** 2010-12-14

**Authors:** Luciana O Araripe, Horácio Montenegro, Bernardo Lemos, Daniel L Hartl

**Affiliations:** 1 Department of Organismic and Evolutionary Biology, Harvard University, Cambridge, Massachusetts 02138, USA

## Abstract

**Background:**

Hybrid male sterility (HMS) is a usual outcome of hybridization between closely related animal species. It arises because interactions between alleles that are functional within one species may be disrupted in hybrids. The identification of genes leading to hybrid sterility is of great interest for understanding the evolutionary process of speciation. In the current work we used marked *P*-element insertions as dominant markers to efficiently locate one genetic factor causing a severe reduction in fertility in hybrid males of *Drosophila simulans *and *D. mauritiana*.

**Results:**

Our mapping effort identified a region of 9 kb on chromosome 3, containing three complete and one partial coding sequences. Within this region, two annotated genes are suggested as candidates for the HMS factor, based on the comparative molecular characterization and public-source information. Gene *Taf1 *is partially contained in the region, but yet shows high polymorphism with four fixed non-synonymous substitutions between the two species. Its molecular functions involve sequence-specific DNA binding and transcription factor activity. Gene *agt *is a small, intronless gene, whose molecular function is annotated as methylated-DNA-protein-cysteine S-methyltransferase activity. High polymorphism and one fixed non-synonymous substitution suggest this is a fast evolving gene. The gene trees of both genes perfectly separate *D. simulans *and *D. mauritiana *into monophyletic groups. Analysis of gene expression using microarray revealed trends that were similar to those previously found in comparisons between whole-genome hybrids and parental species.

**Conclusions:**

The identification following confirmation of the HMS candidate gene will add another case study leading to understanding the evolutionary process of hybrid incompatibility.

## Background

Reproductive isolation is a hallmark of speciation in sexual organisms. When genetically isolated populations have accumulated enough divergence, the hybrid progeny may be sterile due to the disruption of gametogenesis caused by functional incompatibility between factors evolved independently within each population. This scenario characterizes post-zygotic isolation, which is frequently found in pairs of species sharing a recent common ancestor. However, the timing at which reproductive isolation evolves during the process of speciation is somewhat unclear.

An evolutionary scenario of speciation was theorized many decades ago [1,2], but a modern understanding of the speciation process on the molecular level--identifying the so-called "speciation genes"--has just begun to become realistic. A number of studies have succeeded in identifying, at the molecular level, a few genes that may be involved in speciation (in Drosophila: *OdsH *[3,4], *Nup98 *[5], *Nup160 *[6], *Hmr *[7], *Zhr *[8], *Ovd *[9]; in Mus: *Prdm9 *[10], in Xiphophorus: *Xmrk-2 *[11]). These recent data together provide needed insight into the evolution of reproductive isolation, and they largely confirm the traditional view of speciation as an evolutionary process involving multiple genes [12,13]. Moreover, the results imply that speciation is a continuous process that progresses from the occurrence of hybridization with viable hybrids, to hybrid sterility, and ultimately to complete pre-zygotic reproductive isolation. Thus, the existence of multiple reproductive barriers that have accumulated over time is expected [12].

According to the Dobzhansky-Muller model [1,2], genetic incompatibilities arise from negative epistatic interactions between alleles that have appeared within each population and encountered each other for the first time in the hybrid. Thus, sequence differences within at least two loci between two closely related species is a prerequisite for genetic incompatibility. Indeed, the bigger the differences in gene sequence between species, the higher the likelihood that an incompatible sequence variant may have arisen. In this sense, every gene showing rapid evolution might potentially be responsible for generating the incompatibilities in the hybrid of two closely related species.

The abundance of complex epistatic interactions involved in HMS has been recently shown [14-16]. For instance, previous work on the same *D. simulans/D. mauritiana *system used here uncovered several complex epistatic interactions between HMS factors, even though the analysis was restricted to small introgressions in a single background. This limited the results to interactions between factors located not too distant from each other [16]. Nevertheless, the number of genes usually involved and the nature of the epistatic interactions are yet to be resolved.

Although hybrid inviability and/or sterility are the usual outcomes of the disruption of allelic interactions, the sparse data so far accumulated indicate that the underlying nature of the disruptions may vary. The question of whether certain classes of genes are more prone to evolve incompatibilities is still open. Further studies are therefore likely to bring new insights to the topic of speciation. The number and variety of genomic regions found to be involved in some degree of hybrid incompatibility suggest that most of the divergence between species may have accumulated after the rise of reproductive barriers [13,17,18], and indeed several studies have observed an increase in the number of incompatibilities with divergence time [19,20].

Genes causing hybrid inviability may have important housekeeping, developmental, or regulatory functions, whereas genes leading to sterility in hybrids would likely be involved in some aspect of reproduction. Among the genes described so far, three are DNA or chromatin-binding proteins (*OdsH*, *Lhr *and *Hmr*), two are nuclear pore proteins (*Nup96, Nup160*), one is a gene transposition (*JYalpha*), and one is likely to be a small regulatory RNA that suppresses sex-ratio distortion (*Nmy*). As pointed out by Presgraves [21], genomes are not impervious to invasion by selfish elements, and substitutions generated by these leave the same signatures in the genome as beneficial substitutions. Therefore, an alternative to the hypothesis of adaptive evolution is that most of these genes may have evolved as a compensatory response to the effects of deleterious mutations and selfish genes.

In the current work we focus on locating one hybrid-male-sterility (HMS) factor between *D. simulans *and *D. mauritiana *and investigating the nature of the disruption behind it. The HMS *factor 1 *was previously identified by Tao *et al*. [16] as being in a region of 1.4 Mb on chromosome 3 (between molecular markers *Rga *and *Antp*). This is only one of ten factors in chromosome 3 possibly causing hybrid incompatibilities in this pair of species, whose hybrid males are always sterile and females are fertile. Our results show that a fertility shift from quasi-sterile male to a fertile male is associated with a region of 9 kb, in which three complete genes and a portion of one gene are contained. We analyzed the DNA sequence across this interval and found no duplication, deletion, or rearrangement between the two species. However, we observed a handful of divergent sites in the coding sequences of gene *CG17603 *(*Taf1*) and the intronless 576 bp gene *CG1303 *(*agt*), as well as indels present in the 5' UTR of the later and in the intergenic region immediately upstream of its coding sequence. Gene *agt *shows a higher number of non-synonymous (NSS) than synonymous (SS) substitutions, one of the NSS being fixed between *D. simulans *and *D. mauritiana; *the gene also shows a reciprocally monophyletic gene tree. Likewise, the gene tree of *Taf1*, built from the portion of coding sequence included in the mapped interval, unambiguously separates the two species. The other two genes in the region are much more evolutionary conserved and have gene trees that do not differentiate the two species.

## Results

### Mapping of the HMS factor to a 9 kb genomic region

**Figure 1:**
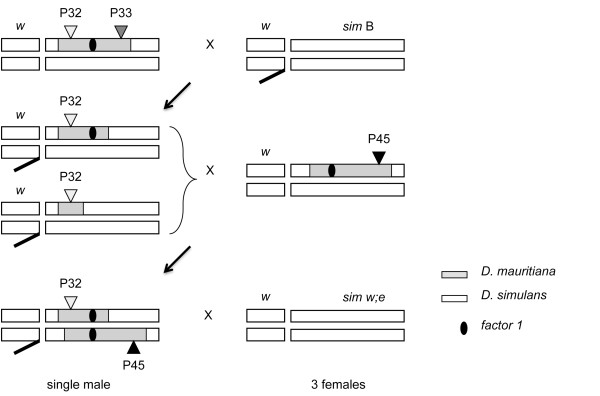
**Mapping design and crosses performed**. Cross scheme used to generate recombinants between two *P-*elements. The second chromosome, marked with *nt *is not shown. Females from 2*P *line 32-33 (see True *et al*. 1996a for *P-*element nomenclature) were crossed to simB males and recombinants were selected by eye color. Recombinants carrying *P*33 had eye color very similar to non-recombinants, which made the selection difficult. Thus, we decided to select only recombinants carrying *P*32 for the fertility tests. The *D. mauritiana *introgression did not cause sterility when heterozygous; so the recombinant lines were crossed to 1*P *line 45.6, whose introgression covers the region where the location of *factor 1 *was predicted. Ten males carrying *P*32 and *P*45.6 from each cross, i.e. homozygotes for the introgression, were selected by eye color and individually crossed to 3 females *w; e*. Fertility was assayed by counting the offspring from each cross up to the 20^th ^day.

Crosses of 2*P*-*cis *females with simB-males generated 536 recombinant males. This number was reached after three runs of crosses, genotyping, and phenotyping. As shown in Figure 1 recombinant males are recognized from the eye color corresponding to *P*32-insert and each bears either the original introgression or one of smaller size that may have been generated by recombination in the mother. In principle, the sizes of the introgressions were not known, and all males were tested by crossing to *P*45.6 (tester stock) and by scoring the fertility of their 2*P *sons (which carry both *P-*elements and introgressions).

**Figure 2:**
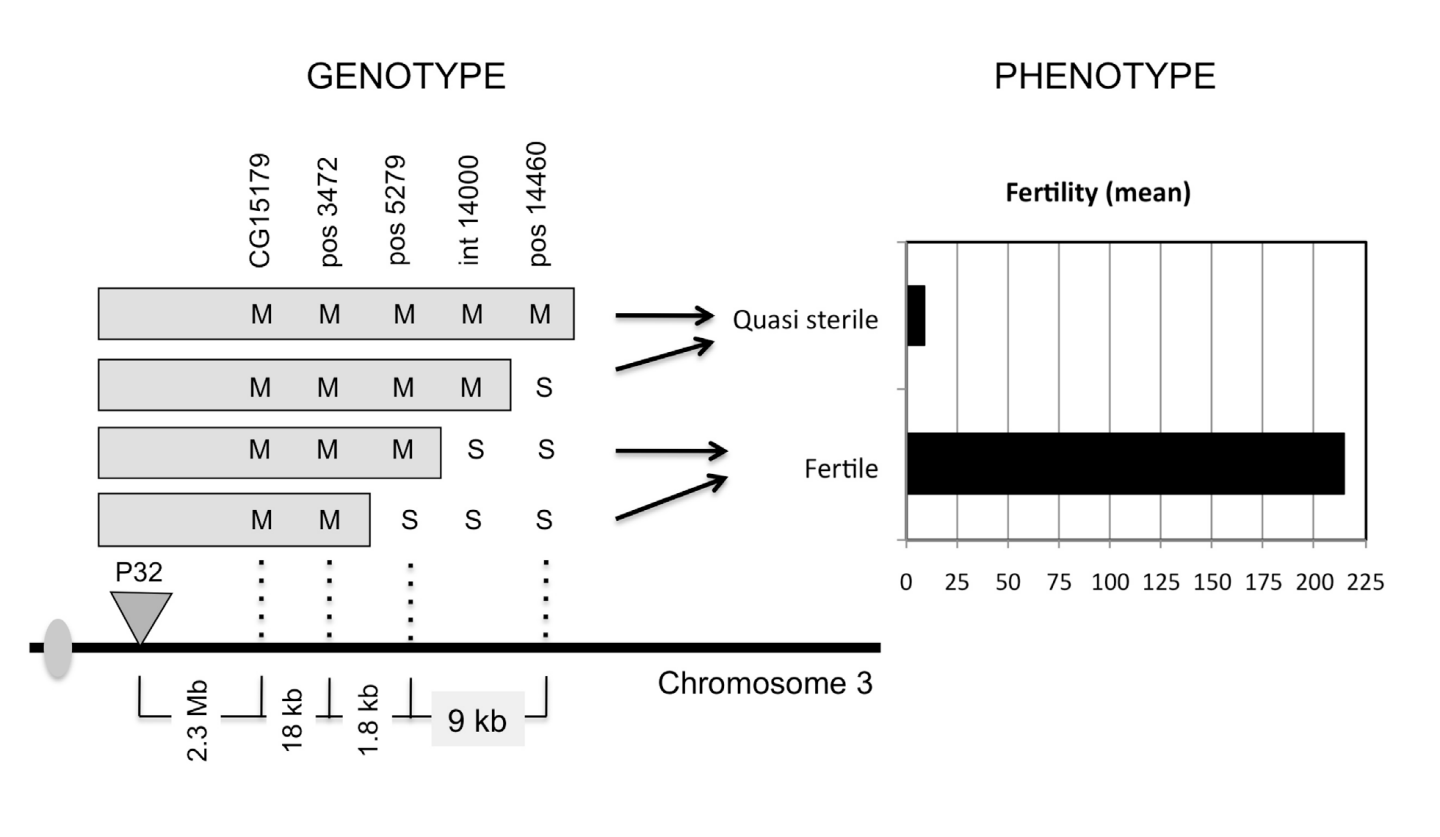
**Scheme showing the final reduction in the region bearing *factor 1***. Summary of results pairing genotype to phenotype. Genotype on each marker site is represented by "M" for *D. mauritiana *and "S" for *D. simulans*. Phenotype was divided in two categories: quasi-sterile gave 9.0 offspring on average (± 2.32) and fertile gave 215.4 offspring on average (± 24.51). The clear-cut localization of *factor 1 *ranges in between markers pos5279 and pos14460, a region of approximately 9 kb.

The screening of the first set of recombinants reduced the region of *factor 1 *from 1.26 Mb (between markers *Rga *and *Antp*) to 372 kb (between markers *CG15179 *and *Antp*) and to chromosomal location 84A1. In the second run, we first genotyped recombinants using the ASO markers on the edge of *factor 1*'s region (*CG15179 *and *Antp*) in order to select for the informative lines and exclude the lines with break points outside of the 372 kb interval. This new effort reduced the region to 170 kb (between markers *CG15179 *and *Dfd*). Finally, the third run helped us locating *factor 1 *within an interval of 20 kb, with four informative recombinant lines within it. The smallest interval between the fertile line *P*32.433 and the quasi-sterile line *P*32.456 is 9 kb (Figure 2 and Additional file 1).

The phenotype corresponding to each genotype class is shown in Figure 2. We summarize the difference in phenotype by showing the mean progeny number for fertile recombinants (215.4 ± 24.51) and quasi-sterile recombinants (9.0 ± 2.32). This represents a reduction of 24-fold in fertility when the 9 kb region of *D. mauritiana *is present in homozygous condition.

The 9 kb region contains three annotated genes: *CG1307*, *CG2358 *(*Spase 18-21*) and *CG1303 *(*agt*). A fragment of gene *CG17603 *(*Taf1*, 3' end representing 44% of the gene and 23% of the transcript) is also included in the region. Additional file 2 shows the genes in the mapped interval. Gene *Taf1 *has molecular functions described as: sequence-specific DNA binding, general RNA polymerase II transcription factor activity, histone serine kinase activity, protein kinase activity, transcription factor activity, and zinc ion binding. Gene *CG1307 *has molecular function described as aminoacyl-tRNA hydrolase activity. *Spase 18-21 *has molecular function described as serine-type peptidase activity. Finally, *agt *has molecular function described as methylated-DNA-protein-cysteine S-methyltransferase activity.

DNA sequencing of the 9 kb region for *D. simulans *and *D. mauritiana *revealed no large duplication, deletion, or chromosomal rearrangement between the species. Across the region we see an even distribution of SNPs and indels, with a clearly higher conservation observed within coding regions (see Additional file 3). Gene *Taf1 *has seven of 16 exons included in the region, *CG1307 *and *CG2358 *have three exons each, and *agt *(*CG1303*) has one single exon. Single-nucleotide differences between species are spread across the whole region, but are especially seen in introns and intergenic regions. Comparing the coding sequences of the genes, both genes *Taf1 *and *agt *show a high density of single-nucleotide differences, whereas no indels were found within exons. The highest divergence between simB and mau12 is seen at the upstream region of gene *agt*. Within a range of 500 bp from the 5' UTR of gene *agt*, we see four small indels, with simB missing a total of 54 bp in relation to mau12 (see Additional file 2).

### Molecular characterization of the candidate interval

**Figure 3:**
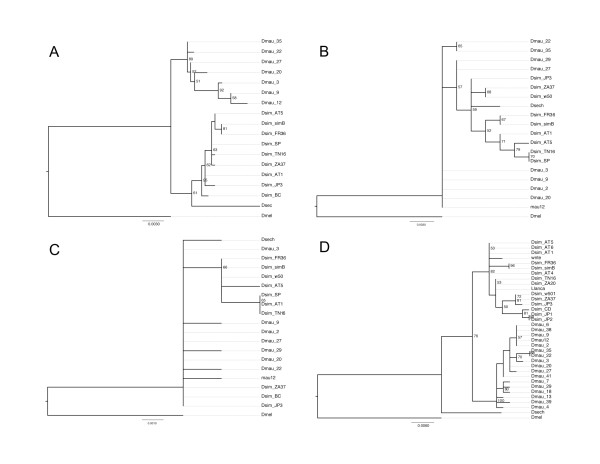
**Phylogenetic trees reconstructed for each gene present in the candidate interval**. Phylogenetic trees reconstructed for each gene present in the candidate interval. For genes (A) *Taf1*, (B) *CG1307*, (C) *CG2358*, and (D) *agt *we analyzed the coding sequence of a number of populations of *D. simulans *and *D. mauritiana*. The species *D. yakuba *was taken as outgroup. The trees for genes *Taf1 *and *agt *showed unambiguous grouping of populations in clades.

A count of the number of substitutions occurring in exons of the four genes for *D. simulans *and *D. mauritiana *allowed an informative analysis of the candidate interval. The results are summarized in Table 1. Genes *CG1307 *and *CG2358 *can be immediately excluded as candidates for the HMS phenotype, as they show no significant variation between species. Gene *CG1307 *shows a total of nine polymorphic sites and one silent substitution fixed between species. We performed a McDonald-Kreitman test, which takes into account a neutral null hypothesis to test whether the ratio of synonymous to non-synonymous substitutions that are fixed between species is consistent with that segregating within species. Fisher's exact test was not significant (one-tailed *P *= 0.5). Gene *CG2358 *(*Spase 18-21*) is very conserved, showing silent polymorphism, but no fixed differences between species (Table 1). Thus, the McDonald-Kreitman test could not be performed. Moreover, the gene trees for *CG1307 *and *CG2358 *do not separate the two species into monophyletic groups (Figure 3B,C).

**Table 1:** Molecular characterization of the candidate interval

	Polymorphism within species	Fixed differences between species	McDonnald-Kreitman	*Ka*	*Ks*	*Ka/Ks*
Taf1	SS = 22	SS = 8	*P *= 0.5	0.0039	0.034	0.114
	NSS = 10	NSS = 4				
CG1307	SS = 5	SS = 1	*P *= 0.5	0.0039	0.020	0.198
	NSS = 4	NSS = 0				
CG2358	SS = 7	SS = 0	NA	0	0.012	0
	NSS = 0	NSS = 0				
agt	SS = 10	SS = 4	*P *= 0.078	0.009	0.047	0.194
	NSS = 15	NSS = 1				

Contrary to *CG1307 *and *CG2358*, the number of substitutions seen for gene *Taf1 *is noticeable. In the relatively small portion of *Taf1'*s coding sequence included in the region (23% of 6.4 kb), we find four non-synonymous substitutions that are fixed between species, in addition to the occurrence of 22 polymorphic sites (Table 1). The gene tree constructed for *Taf1 *unambiguously separates the species *D. simulans *and *D. mauritiana *(Figure 3A). Even though the sequence analysis and gene tree suggest that *Taf1 *may be under rapid evolution, and hence be a good candidate for the hybrid incompatibility, this is a gene that shows relatively high conservation across species of the *D. melanogaster *group (see Additional file 3). Moreover, from among 23 loss-of-function mutations found in a genetic screen of *Taf1*, three were identified as causing female sterility without affecting the fertility of males [22]. The other alleles cause lesions in a variety of structures, including bristles, wings and male terminalia, and some may be lethal.

The other candidate for *factor 1*, the gene *agt*, also shows a high density of single-nucleotide substitutions (Table 1), including 15 that are non-synonymous. Also, the 5' UTR and upstream intergenic region of *agt *show the highest divergence between species, mostly in the form of indels. Conservation in this particular region (*D. simulans *3R: 2517094-2518594) is low across species of the melanogaster subgroup (see Additional file 3) and even between the sister species *D. simulans *and *D. mauritiana*.

Gene *agt *is only 576 bp long (191 amino acids) and has no introns. The protein O-6-alkylguanine-DNA alkyltransferase is involved in the repair of O-6-alkylguanine and O-4-alkylthymine in DNA, and in most organisms it attenuates the cytotoxic and mutagenic effects of certain classes of alkylating agents. Between *D. simulans *and *D. mauritiana *the coding region of gene *agt *shows a higher number of non-synonymous (NSS) than synonymous (SS) substitutions: *D. simulans *has 13 segregating sites with seven NSS, whereas *D. mauritiana *has 12 segregating sites with eight NSS. It is important to highlight that one non-synonymous substitution is fixed between species (Table 1). However, Fisher's exact test was not significant (one-tailed *P *= 0.078).

In addition to its degree of sequence divergence, *agt's *gene tree is perfectly consistent with the separation of *D. simulans *and *D. mauritiana *into monophyletic groups (Figure 3D), in contrast to the gene trees of *CG1307 *and *CG2358*. In fact, it is expected that genes involved in speciation will reflect more accurately the phylogenetic history of closely related species [23,24], and this indeed has been shown to be the case for another gene causing hybrid sterility, *OdsH *[25].

The non-silent difference between species at position 361 of *agt'*s coding region is also a variable site when other pairs of species are considered (Table 2). *D. erecta *and *D. yakuba *have GAT (aspartic acid) in position 361, *D. melanogaster *has CAT (histidine), *D. sechelia *has TAT (tyrosine), *D. mauritiana *has AAT (asparagine), and *D. simulans *has GAT (aspartic acid) like the outgroup *D. yakuba*. This specific change may suggest a precise location for the origin of the hybrid incompatibility.

**Table 2:** Amino acid replacement in position 361 of the candidate gene agt

Species	Codon at position 361	Amino acid
*D. simulans*	GAT	Aspartic acid (Asp)
*D. mauritiana*	AAT	Asparagine (Asn)
*D. sechelia*	TAT	Tyrosine (Tyr)
*D. melanogaster*	CAT	Histidine (His)
*D. yakuba*	GAT	Aspartic acid (Asp)
*D. erecta*	GAT	Aspartic acid (Asp)

Our results point to genes *Taf1 *and *agt *as good candidates for the hybrid male incompatibility *factor 1 *mapped to the 9 kb region herein reported. However, the fact that gene *Taf1 *is not entirely represented in the mapped interval and, in *D. melanogaster*, affects the fertility of females and not males when disrupted, makes this gene a less attractive candidate for causing the male-sterile phenotype than it might otherwise be.

### Complex epistasis between HMS and the genomic background

**Figure 4:**
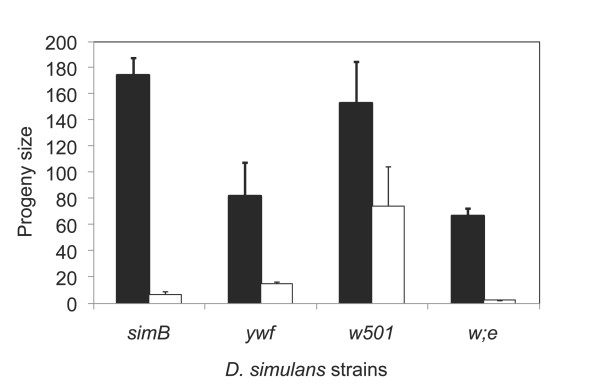
**Interaction of *factor 1 *with different genetic backgrounds of *D. simulans***. Progeny size resulting from the presence of two copies of *D. mauritiana *introgression *P*32.75 (black columns) and *P*32.110 (white columns) in different *D. simulans *backgrounds. The HMS *factor 1 *is present in homozygous condition in *P*32.110 and clearly causes a reduction in fertility for every background tested. Vertical bars indicate the standard errors.

We have tested whether the HMS *factor1 *was similarly expressed in three different genomic backgrounds. We find that significant variation in fertility is observed when other strains of *D. simulans *are used. In order to establish stocks for this experiment, males from recombinant lines *P*32.75 and *P*32.110, as well as males from the tester stock *P*45.6, were crossed to females from three different strains of *D. simulans*: w501, *w*; *e*, and *ywf*. Male progeny with colored eyes were backcrossed to virgin females from the respective *D. simulans *strain for five more generations. Because no recombination happens in males of most species of *Drosophila*, at this point we expected that each original recombinant chromosome would be intact, whereas the rest of the genome would have been largely replaced by the background strain. Females from each background were then crossed to males from the corresponding *P*45.6 line and the 2*P *male progeny tested for fertility. The fertility tests were done as described in Material and Methods: each male 2*P *was crossed to three females *w*; *e *and the progeny were counted. The results are shown in Figure 4. The *w*; *e *background gives a 30-fold difference in fertility between the fertile line *P*32.75 (*factor 1 *absent) and the quasi-sterile line *P*32.110 (*factor 1 *present in homozygosis). The smallest effect of *factor 1 *was seen in the w501 background (2-fold), even though the difference in progeny size is still highly significant (*P *< 0.01).

### Gene expression analysis

Patterns of gene expression may also give clues as to the molecular nature of *factor 1*. For this, we began by investigating the tissue-specificity of the four genes contained within the introgressed region using publicly available data for *D. melanogaster *(FlyAtlas [26]). Gene *Taf1 *is expressed at low and similar levels in all tissues; expression level in testes is basal and reported to be half that for ovaries. Gene *CG1307 *has a developmentally homogeneous expression pattern that appears to be restricted to tubule and hindgut tissues. Taken together with its lack of evolutionary variability, this pattern appears sufficient to rule it out as the cause of the HMS herein observed. Gene *CG2358 *is ubiquitously expressed although levels vary greatly across tissues. Its highest expression level is in salivary glands. Moreover, its high sequence conservation across species allows us to rule it out as a cause for HMS. Finally, gene *agt *is expressed at low levels and just above the detection limit in various tissues. Importantly, in the male accessory gland, *agt *is expressed in levels at least two times higher than most of the other tissues. Male accessory glands are required for sperm storage and male fertility.

**Figure 5:**
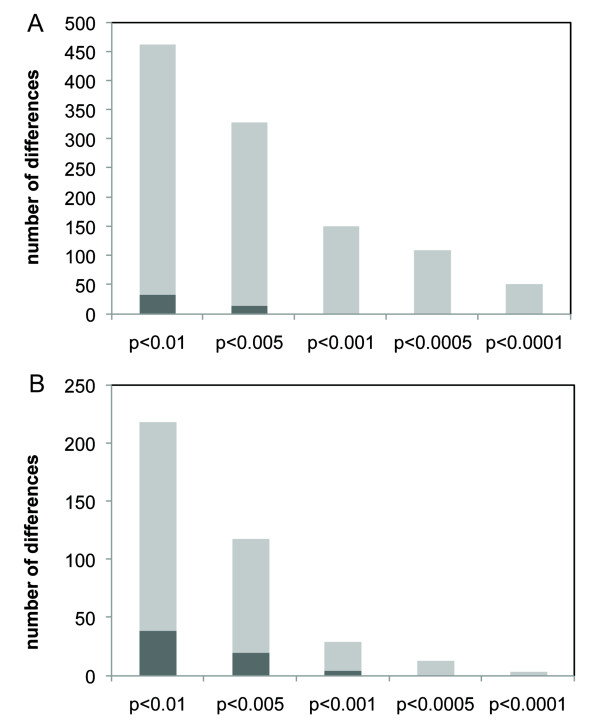
**Average gene expression differences between one fertile and 4 non-fertile lines**. (A) Average number of genes down regulated in non-fertile lines when compared to one fertile line. (B) Average number of genes up regulated in non-fertile lines when compared to one fertile line. Dark gray bars indicated the number of genes expected in each class of comparison and light gray bars indicate the observed numbers.

Microarray analysis helped identifying the molecular correlates of the hybrid male sterility and showed a substantial number of gene expression differences associated with the non-fertile phenotype relative to the fertile phenotype (Figure 5). First, we found 932 genes whose expression varies between the fertile and non-fertile lines (*P *< 0.01, *FDR *< 0.10, see Additional file 4). Furthermore, we found that 157 genes (FDR < 0.05) show concordant gene expression differences in all four non-fertile lines relative to the fertile line (see Additional file 5), with 124 genes similarly down-regulated in all four non-fertile lines and only 33 genes similarly up-regulated in the same lines - see also Figure 5. Importantly, the genes affected are randomly scattered throughout the genome with only 5 down-regulated targets contained within the introgressed segment. Altogether, the genes differentially expressed between lines underscore the regulatory networks that are disrupted by *factor 1 *or other divergent elements in the mapped region. Interestingly, while the genes detected show a broad distribution of functional classes, we detected a statistically significant enrichment for genes whose functions are associated with spermatogenesis (*P *= 0.002, Fisher's exact test). Accordingly, we observed 19 downregulated genes and 11 upregulated genes in non-fertile lines and belonging to the gene ontology category of "spermatogenesis".

## Discussion

The history of divergence of *D. simulans *and *D. mauritiana *from a common ancestor dates from ~0.3 million years ago [27] and likely happened through the common mechanism of allopatric speciation [28]. The distribution of the two species overlapped recently, at some point around 24,000 years ago, as evidenced by an introgression of *D. simulans *mtDNA into *D. mauritiana *[29]. The rise of reproductive isolation in this system has been the object of several studies. Hybrid male sterility (HMS) loci have been found mainly on the X chromosome (reviewed in Wu & Hollocher [30]), but Tao *et al*. [16] have described the occurrence on the 3^rd ^chromosome of 10 HMS factors by genetic mapping, among a total of 19 quantitative trait loci (QTL) in this chromosome that may be involved in hybrid incompatibilities.

The use of QTL mapping to identify the genomic region responsible for the expression of a complex phenotype has been extensive in several organisms. Because hybrid sterility as a complex trait may result from disruptions in varied genes and genetic interactions, the use of a mapping approach that mixes two genomes in equal proportions is very likely to give no fertile individuals. Nevertheless, the use of introgressions of one species in the genomic background of another species has been effective in the search for the molecular basis of HMS. Since introgressions vary in size and represent a very small proportion of the hybrid genome, they may or may not cover the factor responsible for the disruption and a range of fertility phenotypes results. For this reason, introgressions have been classically used in the mapping of HMS [16,30-32].

On the other hand, the mapping through introgressions may not detect small HMS sites when complex epistasis is present. Another limitation is that genetic rearrangements may show incompatibilities that do not really exist, for instance, when an event of transposition involving the introgression happens, we may lose an essential gene in one of the species and this can generate sterility or inviability that is not associated with hybrid incompatibilities [33]. In our case the use of introgressions was facilitated by the fact that previous work had already established the parental lines. In addition, QTL mapping had been previously done in the same region [16].

### HMS expression depends on genomic background

In previous work, two of the HMS factors located on chromosome 3 were fine-mapped and characterized at the molecular level [16,34,35]. These factors have a large effect on HMS, but several other factors of small effect may be acting additively [36] or epistatically [15,37] in generating the hybrid incompatibility. These results support the view that that HMS is often a polygenic trait.

The polygenic nature of the hybrid sterility also accounts for the incomplete penetrance and variable phenotypic expression in each background. We tested three different backgrounds besides the stock simB used for the mapping. We find the same qualitative result, a reduction in fertility due to the presence of two *D. mauritiana *alleles of *factor 1*. However, the magnitude of this difference varies according to the background. In the background of line *w*; *e*, the presence of *factor 1 *when homozygous causes fertility to drop 30 fold, whereas in the w501 background fertility drops only twofold (Figure 4). This contrast indicates that there may be a complex network of negative epistatic interactions causing HMS. Thus, the effect of hybridization may depend on several factors that frequently vary among different lines of each species.

### Regulatory effects of HMS

Information on gene expression is provided by the FlyAtlas [26] and refers to expression in different tissues of *D. melanogaster*. All genes contained in our interval are available in the dataset. Gene *CG2358 *(*Spase 18-21*) shows enrichment in salivary glands, male accessory glands, and larval salivary gland. Expression of this gene is up regulated in these tissues. Among the other three genes, *agt *shows a twofold enrichment in male accessory glands.

Recent studies of gene expression in hybrids have found that the misexpression of genes involved in spermatogenesis may cause sterility in hybrids [38,39]. Most of these genes are underexpressed in the hybrids relative to the parental species [20,40], and this finding might reflect a disruption of gene interactions that are particular to each species. These results come from work where whole-genome hybrids were compared to both parental species, which is a situation different than our study. Here we used lines that bear a hybrid region considerably smaller (3R: 1,468,434..7,938,322), in the background of *D. simulans *(more specifically line simB). Most importantly, the segment that differs between the fertile and non-fertile lines is only ~1.4 Mb and contains only 174 known protein coding genes. Yet we find results qualitatively similar: the average number of down regulated genes in hybrids was more than double the average number of up regulated genes and both values were much greater than expected (Figure 5). Importantly, genes belonging to the Gene Ontology category of spermatogenesis are preferentially affected, with 30 targets showing differential expression.

Artieri *et al*. [41] showed that underexpressed genes in hybrids appear to evolve more rapidly than genes expressed normally in hybrids. This fact contributes to the idea that rapid evolution reduces gene similarity and potentially causes genetic incompatibilities.

### Molecular evolution of HMS

The fine mapping described here defined a region as small as 9 kb that includes the candidate causing the great reduction in fertility in hybrid males of *D. simulans *and *D. mauritiana*. None of the genes present in this region are clearly involved in spermatogenesis, although signals of rapid evolution are present and help to suggest a candidate for *factor 1*. Gene *agt *is a small gene (576 bp) with high polymorphism within species and a number of nucleotide substitutions between species (Table 1). Among the fixed substitutions, four are synonymous and one is non-synonymous. Moreover, the region where *agt *is present shows very low conservation.

Similarly to *agt*, the gene *Ovd *(*GA19777*), recently described by Phadnis and Orr [9], lacked strong evidence of non-neutral evolution but proved to be the best candidate for the hybrid incompatibilities between the subspecies *D. pseudobscura pseudobscura *and *D. pseudobscura bogotana*. The authors found that this gene is involved in causing both segregation distortion in the F_1 _and hybrid male sterility.

Identifying the normal function of a candidate gene within the parental species is of great interest when investigating the basis of hybrid incompatibilities. So far, no particular function can be attributed to genes involved in speciation. Some are enzymes, some are transcription factors, and others are structural proteins [42]. One possible explanation for the involvement of these varied classes of genes in reproductive isolation is that genetic substitutions accumulate over time, ultimately leading to enough divergence to cause genetic incompatibilities [7,21]. Nevertheless, the most common characteristics of genes involved in hybrid male sterility are signals of rapid evolution and positive selection within species [12,42].

One candidate for *factor 1 *in our study, gene *agt*, is reported as being involved in the repair and attenuation of the toxic and mutagenic effects of certain alkylating agents. Kooistra *et al*. [43] showed that the expression of *agt *suppresses transition mutations (G:C to A:T and vice-versa) *in vivo*. At the molecular level, *agt *is involved in methyltransferase activity. Apparently, this function does not have any clear association with reproduction for its disruption to lead to male sterility. Nevertheless, the gene may have as yet unidentified functions, or its enzymatic function may be deployed in some manner essential to hybrid male fertility.

In the mouse, the recently identified speciation gene *Prdm9 *is known to encode a meiotic histone H3 methyltransferase [10,44]. In the parental species, *Prdm9 *activates genes essential for meiosis and thus is essential for reproduction. The disruption of this function in hybrids of *Mus m. musculus *and *Mus m. domesticus *leads to male sterility, similarly to the phenotype of the *Prdm9^-/- ^*mutants. Similarly to *Prdm9 *and *Ovd*, the earlier identified HMS gene (*OdsH*) was recently reported to also encode a protein with putative DNA binding domain [45]. Thus, proteins that bind to chromatin and have possible regulatory roles may represent the most common class of factors whose disruption leads to hybrid incompatibilities. The gene *Taf1 *also functions as sequence-specific DNA binding protein and shows transcription factor activity. The fact that *Taf1'*s function corresponds to what is described for most of the genes involved in hybrid male sterility may, *per se*, suggest this as a plausible candidate gene for *factor 1*.

Across the Drosophila phylogeny, *agt *has undergone substitutions more often than other functional genes in the candidate region we mapped. Strikingly, substitutions in position 361, cited in the previous section, occurred in every clade since the split of *D. melanogaster*, and all lead to amino acid substitution. This information may indicate that *agt *is evolving rapidly and systematically changing with every branching event. Substitutions in position 361 are fixed within species and may be the key difference leading to the drop in fertility seen in hybrid males. Another observation is that *agt *shows unambiguous sorting of the three species of the simulans clade (Figure 3), as also observed for *OdsH*, another gene involved in hybrid incompatibility between *D. simulans *and *D. mauritiana *[25]. Ongoing experiments focus on confirming the role of genes *Taf1 *or *agt *in causing the HMS via germ-line transformation rescue.

## Conclusions

Our results suggest two candidate genes possibly leading to HMS between *D. simulans *and *D. mauritiana*. The mapping of such a complex phenotype down to a 9 kb region and to identifying candidate genes is an important achievement for the field and contributes to the knowledge of what classes of genes may cause HMS when disrupted in hybrids. Further experiments will investigate the functional role of *Taf1 *and *agt *in causing the decrease in fertility.

## Methods

### *Drosophila *stocks

*D. simulans*: (1) simB: *w*; *nt*; III (*white*; *net*; third chromosome homozygous and isogenic to that of line 13w 1 × 1JJ). The construction of 13w 1 × 1JJ and simB was described earlier [34,46]; (2) sim *w*; *e *(*white*; *ebony*). All the stocks were provided by J. Coyne and maintained in the laboratory for several generations.

*D. mauritiana*: *w *(*white*); *P*[*w*^+^], lines with independent *P-*element insertions on the third chromosome [47]. The *P*[*w*^+^] inserts are semi-dominant markers with position effect, i.e., the wild form of *white *carried in the *P-*element produces an eye color between yellow and red, depending on the location of the *P*-insert.

The choice of lines to use for the mapping of *factor 1 *was based on the work of Tao *et al*. [16,34]. In that study hybrid lines between *D. simulans *and *D. mauritiana *were constructed. After several generations of backcrossing to the simB line (described above) and selecting for the colored-eye progeny, a piece of *D. mauritiana*'s 3^rd ^chromosome of varying size was introgressed into the genome of *D. simulans*. A total of 231 introgression lines were created from a set of 28 *D. mauritiana *lines bearing one copy of *P*[*w*^+^] independently inserted in the 3^rd ^chromosome. Details of the introgression scheme are in Tao *et al*. [[34], Figure 4], as well as the names given for the introgression lines.

Three lines composed of a simB background and one *P*[*w*^+^]-tagged *D. mauritiana *introgression on the third chromosome were used for the mapping. The creation of these lines is described elsewhere [34,47].

### Genetic mapping

Lines *P*32.8 (yellow eye) and *P*33.3 (red eye) were chosen for having *P-*element inserts flanking *factor 1 *[47,48]. Two generations were necessary to construct a heterozygote line with both *P-*inserts in *cis: P*32.8 females and *P*33.3 males generated a proportion of offspring with both *P-*inserts in *trans*, which could be distinguished from the others by their dark-red eyes. Females with inserts in *trans *were crossed to simB males, and the darker-eye offspring selected again as bearing *P*32 and *P*33 inserts in *cis *(Figure 1). The 2*P *construct carries a *D. mauritiana *introgression that covers the region where *factor 1 *had previously been located [31]. Other two factors identified as possible HMS (#9 and #10 - [16]) were previously located in regions covered by the 2*P *construct we generated. However, the existence of these factors is not a source of influence on our results, as factor #9 is always present in every *P*32 recombinant line used here and factor #10 is never included in the fine mapping (i.e. when we focused on recombinant lines bearing small introgressions). Thus, only the presence or absence of *factor 1 *may be associated to fertility or sterility.

The cross of 2*P *females to simB males generates single-*P *recombinants, which can be recognized by an eye color that is lighter than in the original *P *lines; these carry *D. mauritiana *pieces of different sizes (Figure 1). The ideal 2*P *design uses recombinant lines having either of the *P*-inserts from the parental lines, thus flanking *factor 1 *from both sides. However, in the present case, a reliable separation of *P*33 recombinants from 2*P *non-recombinants based on eye color was not possible. We thus decided to establish lines only from *P*32 recombinants.

Each recombinant male was crossed to five females of simB in order to establish recombinant lines bearing heterozygous *D. mauritiana *introgressions. Because a single copy of the introgression does not harm male fertility, these lines were maintained through males × simB females in every generation. Moreover, since Drosophila males do not have recombination, the transmission through males assures the integrity of introgressions, and hence the perpetuation of the recombinant line. Males from stable recombinant lines were then taken for genotyping (assessment of introgression length) and fertility tests.

### Fertility assay

Although the lack of recombination in Drosophila males can be very convenient for designing genetic experiments, it allows spontaneous mutations to accumulate through Muller's ratchet. Some of these mutations may cause sterility when in homozygosity. The frequency of spontaneous sterility was estimated as ~1.5% [34,49] which is capable of blurring the fertility tests. In order to circumvent this concern and bring *factor 1 *to a homozygote state, we generated trans-heterozygote males from two independently raised *P*[w^+^] stocks, i.e., males from *P*32 recombinant lines were crossed to females from *P*45.6 (named the tester stock), and the male offspring with this combination of *P*-inserts (dark-red eyes) were selected for fertility tests (Figure 1). In this way, no spontaneous mutation occurring in the original *P*32.8 or *P*45.6 will be homozygous, whereas *factor 1 *may or may not be homozygous depending on the size of the introgression in each case.

Ten *trans*-heterozygote males were selected from each cross for the fertility tests. The typical fertility analysis used in previous work is based on the number of motile sperm present in seminal vesicles [50]. Here we follow the assay by Tao *et al*. [31,34], which is based on counting viable offspring derived from *trans*-heterozygote males. This is a more quantitative method that allows us to separate by sex (in order to investigate the occurrence of sex-ratio distortion) and eye color. Each of 10 *trans*-heterozygote males was crossed to three virgin females of *D. simulans w*; *e *for seven days. After this period, females were discarded and males were collected for single-fly genotyping.

Offspring were counted up to the 20^th ^day and males classified as fertile or quasi-sterile. We observed that two copies of *factor 1 *cause either a severe drop in fertility or complete sterility. Recombinant lines were classified as quasi-sterile when their *trans*-heterozygote males had on average zero to 30 offspring. This range was empirically chosen, as outside this range the fertility jumps to an average of 120 offspring or more. A negligible number of males had progeny numbers between these two categories and were removed from the analysis.

### Single-fly genotyping

After the seven-day mating period with *w*; *e *females, *trans*-heterozygote males were collected and placed one in each well of 96-well plates. Grinding solution was added in each well (40 μl of 10 mM Tris pH 8.2, 1 mM EDTA, 25 mM NaCl, 0.2 mg/ml Proteinase K) and flies were homogenized. The plates were incubated in 65° for 30 min, 95° for 2 min and chilled on ice briefly before being stored at -20°.

The genotyping made use of molecular markers from various sources. First, allele-specific oligonucleotide markers previously developed (ASO [51]) were used as external markers in order to delimitate the region. We then designed additional ASO markers as the genetic dissection of the HMS region progressed. The ASO probes are pairs of 15-mers that recognize the same sequence, but carry one or more SNPs (single nucleotide polymorphisms) between *D. simulans *and *D. mauritiana*. The steps for designing ASO probes are described in detail by Tao *et al*. [34]. In their work, primers were designed using the genome of *D. melanogaster *as template. However, in the present work we could take advantage of the genome project completed for *D. simulans*, as well as some regions of *D. mauritiana *obtained from 454 Life Sciences sequencing carried out at the Genome Center at Washington University in St. Louis.

Other markers were based on PCR success/failure using species-specific primers and PCR products with species-specific sizes. In the first case, triads of primers were designed in order to have one of them, either forward or reverse, annealing perfectly to both species, and a pair showing species-specific annealing. Additional file 6 lists the molecular markers used, as well as the primers, probes, and experimental conditions for their use. All oligonucleotides were designed using the online tool of Primer 3 http://frodo.wi.mit.edu.

During the final mapping step, we sequenced 20 kb spanning the region bearing *factor 1 *for simB, mau12, and *w*; *e*. The 20 kb region was split into seven ~3 kb-pieces in order to facilitate PCR reaction and downstream methods. We extracted DNA from ~10 flies of each stock using DNeasy (QIAgen). PCR reaction was performed using TaKaRa LA Taq (Takara Bio Inc.) and the protocol: 94° for 1 minute; 30 cycles of 94° for 15 seconds, 55° for 30 seconds, 68° for 5 minutes and extension in 72° for 10 minutes. PCR products were cleaned with ExoSAP-it (USB). In total, 36 pairs of primers were used to sequence the seven pieces. This coverage provided a complete set of SNPs and indels and served as a reliable and straightforward source for genotyping.

### Molecular characterization of the candidate interval

We sequenced the 20 kb extent of the candidate region for the lines simB, *w*; *e*, and mau12. For a length of 1 kb encompassing the coding and flanking regions of gene *agt *(*CG1303*), an additional 15 strains of *D. simulans *from different locations across Africa and the Americas, and 17 strains of *D. mauritiana *collected in 2006 (kindly provided by Dr. Maria Margarita Ramos), were sequenced. Regions of 2.2 kb (*Taf1*), 3 kb (*CG1307*) and 2.4 kb (*CG2358*) were sequenced for a subset of 8 strains of *D. simulans *and 8 of *D. mauritiana*. Contig assembly was performed with Sequencher 3.0 (Gene Codes Corporation, Ann Arbor, MI, USA) and alignment was performed using ClustalW software [52].

Molecular genetic analyses, including the McDonald-Kreitman test, were performed with DnaSP [53]. Phylogenetic tree reconstruction was performed for each gene's coding region separately, using maximum likelihood with phyML software [54], after running jModelTest [55] to determine the best fitting model to each alignment. The phylogeny obtained for each gene was used in the detection of positive selection with the software PAML [56].

### Gene expression analysis

**Figure 6:**
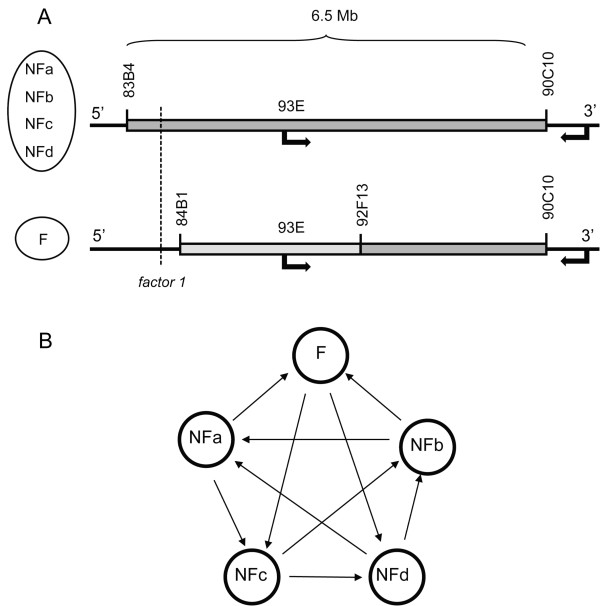
**Microarray design comparing fertile and non-fertile lines**. (A) Design for the microarray experiment comparing gene expression in one fertile and four non-fertile lines. The fertile line bears an introgression that may or may not include the light-gray region, but definitely excludes the portion where *factor 1 *is located (83B4-84B1). Black arrows indicate inversion previously known in *D. simulans *and *D. mauritiana*, in relation to *D. melanogaster*. (B) Microarray design showing all the comparisons among lines. In our design, four independently obtained lines with non-fertile phenotypes were compared with a fertile reference. These lines only differ in the presence of a small segment of *D. mauritiana *where *factor 1 *is present. In the fertile line this segment is present in heterozygosity with the homologous segment from *D. simulans*, whereas the segment is homozygous *D. mauritiana/D. mauritiana *in the four non-fertile lines.

Genome-wide microarray analyses of gene expression of fertile and non-fertile lines were performed. The lines used in this essay were recombinant lines generated from an early step of the mapping process, when introgressions were covering a large region of chromosome 3R. Five recombinant lines were chosen and crossed to the tester stock *P*45.6 in order to bring *factor 1 *to a homozygous condition. Lines #96 and #102 were completely sterile, but were genotyped as having introgression of same size as quasi-sterile lines #143 and #188. For this reason, these lines were merged in the same group, non-fertile, and compared to the only normally fertile line #225 (Figure 6A). Line #225 bears a smaller introgression generated by recombination in the mother. The breakpoint excluding *factor 1 *from this line was located in between markers *Antp *and *CG31195*, but not precisely determined at this early step of the mapping. Thus, the introgressions present in these lines are identical at their 3' end but differ at the 5' end. Except for these differences in the amount of introgressed material and the consequential presence or absence of *factor 1 *and other elements within the introgressed region, these lines are genetically identical.

Microarrays were ~18,000-feature cDNA arrays spotted with *D. melanogaster *cDNA PCR products. Total RNA was extracted from whole flies using TRIzol (Life Technologies) and microarray analyses were performed with standard protocols previously described [57]. Using RNA from testis would focus the results on the specific effects of *factor 1 *on spermatogenesis, but on the other hand, would not give any information about the effects of *factor 1 *on genes that are exclusively expressed in other tissues. The microarray design implemented in this study is shown in Figure 6B.

The cDNA synthesis, the labelling with fluorescent dyes (Cy3 and Cy5), and the hybridization reactions were carried out using 3DNA protocols and reagents (Genisphere). Slides were scanned using an Axon 4000B scanner (Axon Instruments) and GenePix Pro 6.0 software. Foreground Fluorescence of dye intensities was normalized by the Loess method in the R Limma library. Stringent quality-control criteria were used to ensure reliability of foreground intensity reads for both Cy5 and Cy3 channels. These conservative criteria were the following: (([F635Median - B635] > 4*[B635 SD] OR [F532 Median - B532] > 4*[B532 SD]) AND ([% > B635+2SD] > 70 OR [% > B532+2SD] > 70) AND ([F635 % Sat.] < 45 AND [F532 % Sat.] < 45) AND ([B532 Median] < 4*[B635 Median] AND [B635 Median] < 4*[B532 Median]) AND ([Sum of Medians (635/532)] > 100) AND ([SNR 635] > 2 AND [SNR 532] > 2) AND ([Rgn R2 (635/532)] > 0.5) AND ([Circularity] > 0.45)), where F532 and F635 denote the foreground fluorescence intensities, B532 and B635 denote the background fluorescence intensities, SNR 532 and SNR 635 denote signal to noise ratio for Cy3 and Cy5, respectively. Rgn R2 and circularity denote the spot specific coefficient of determination and spot specific circularity as calculated by the GenePix software.

The significance of variation in gene expression due to the introgressed segment causing HMS was assessed with linear models in Limma and with the Bayesian Analysis of Gene Expression Levels (BAGEL). FDRs were estimated based on the variation observed when randomized versions of the original dataset were analyzed.

The data discussed in this publication have been deposited in NCBI's Gene Expression Omnibus [58] and are accessible through GEO Series accession number GSE25339 http://www.ncbi.nlm.nih.gov/geo/query/acc.cgi?acc=GSE25339.

## Authors' contributions

LOA carried out the design of the study, genetic crosses, analysis of phenotype and genotype, and drafted the manuscript. HM performed the experiment in different genetic backgrounds, helped with analyses, and gave suggestions on the draft. BL designed, performed and analyzed the microarray experiment, and helped to draft the manuscript. DLH participated in the design and coordination of the study, and gave suggestions on the draft. All authors read and approved the final manuscript.

## Supplementary Material

Additional file 1 **Recombinant lines and phenotypes at the final step of mapping *factor *1**. Detailed localization of *factor *1 according to four recombinant males showing introgression of similar sizes and different phenotypes. Only one chromosome is shown for each male. Recombinant break points were identified based on SNPs within genes (large font) or in the intergenic region (small font). Phenotype is given by the mean progeny size and standard error below each chromosome. The mean is based on 10 homozygous males from each recombinant line (see Methods). Finally, we show the position of *factor *1 according to the annotated *D. simulans *genome. Click here for file

Additional file 2 **Graphic scheme of the region where *factor 1 *is located**. (A) Graphic scheme showing the 9 kb mapped region and the genes found within it (gene span and mRNA). Note that only seven of *Taf1'*s 16 exons are contained in the region. The arrows show approximate location and relative size of indels found in the upstream region of gene *agt*. (B) alignment of a portion of the upstream region of gene *agt *for different populations of *D. simulans *and *D. mauritiana*. Click here for file

Additional file 3 **Inter-species conservation across the mapped region**. Graph from UCSC alignments showing the degree of conservation across species in *D. melanogaster *group and close species. The reference sequence represents the species *D. melanogaster*. A range of 20 kb is shown. Coding regions show much higher conservation than introns and intergenic regions. However, the coding region of gene *agt *shows low conservation across species (yellow stripe). Click here for file

Additional file 4 **List of genes showing misexpression in at least one of the non-fertile lines**. Click here for file

Additional file 5 **List of genes with misexpression congruent in all four non-fertile lines**. Negative values mean that genes were down regulated in the non-fertile lines in relation to the fertile one and positive values mean up regulation in the non-fertile lines. The 5 down regulated genes contained within the introgressed segment are shown in red. Click here for file

Additional file 6 **List of molecular markers used for mapping *factor 1***. Click here for file
