# Supplementary figures and images for: Fine-scale genetic mapping of a hybrid sterility factor between Drosophila simulans and D. mauritiana: the varied and elusive functions of "speciation genes"
Source: BMC Evol Biol. 2010 Dec 14;10:385. doi: 10.1186/1471-2148-10-385 (PMC3020225; doi:10.1186/1471-2148-10-385)

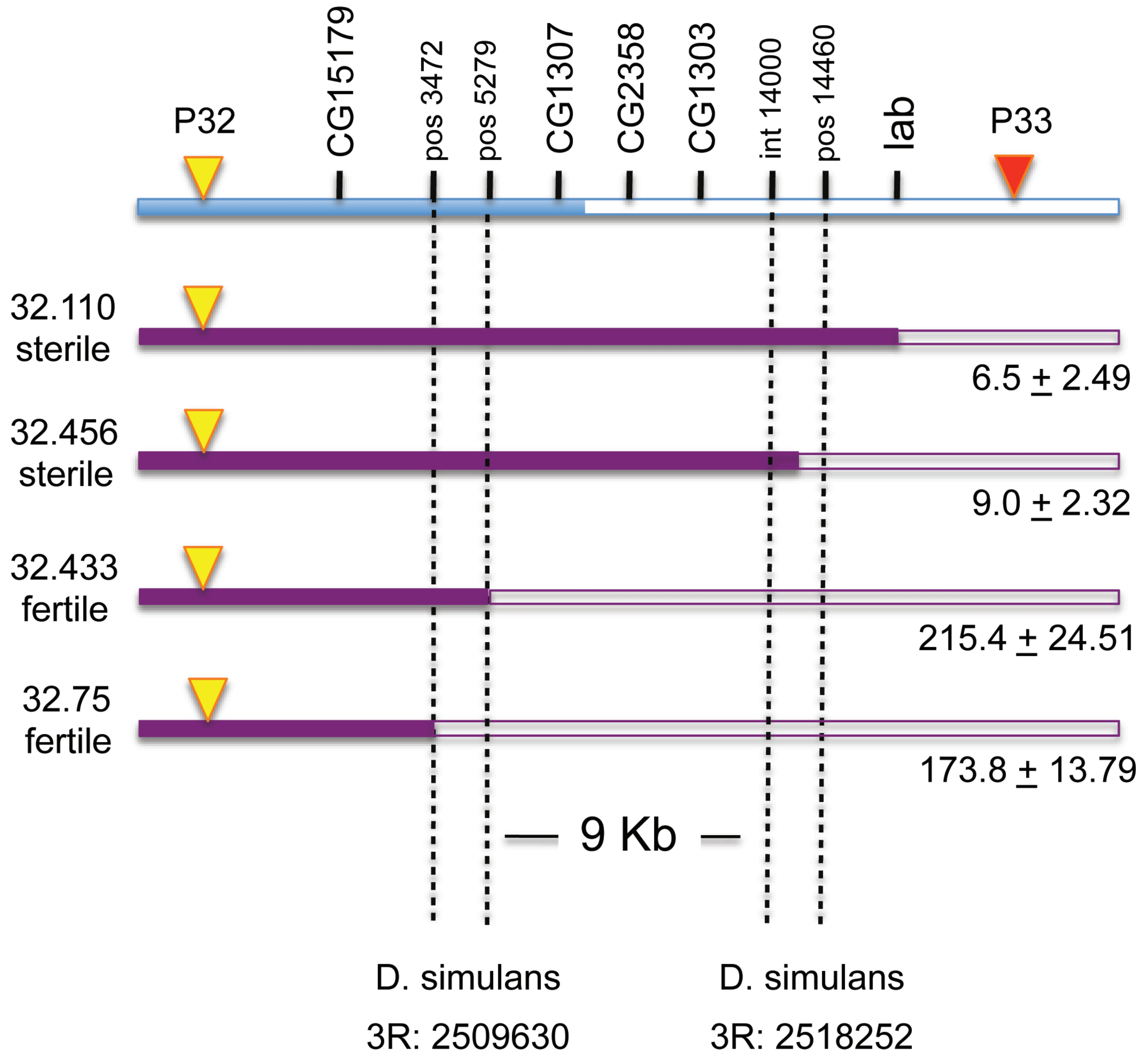

Supplement: Additional file 1 — Recombinant lines and phenotypes at the final step of mapping factor 1. Detailed localization of factor 1 according to four recombinant males showing introgression of similar sizes and different phenotypes. Only one chromosome is shown for each male. Recombinant break points were identified based on SNPs within genes (large font) or in the intergenic region (small font). Phenotype is given by the mean progeny size and standard error below each chromosome. The mean is based on 10 homozygous males from each recombinant line (see Methods). Finally, we show the position of factor 1 according to the annotated D. simulans genome. [file 1471-2148-10-385-S1.PDF]

A

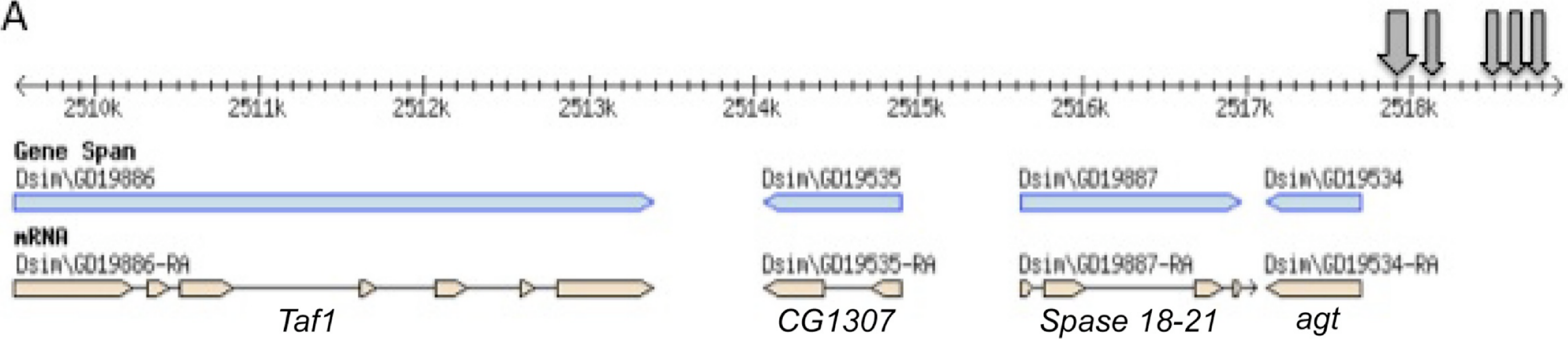

B

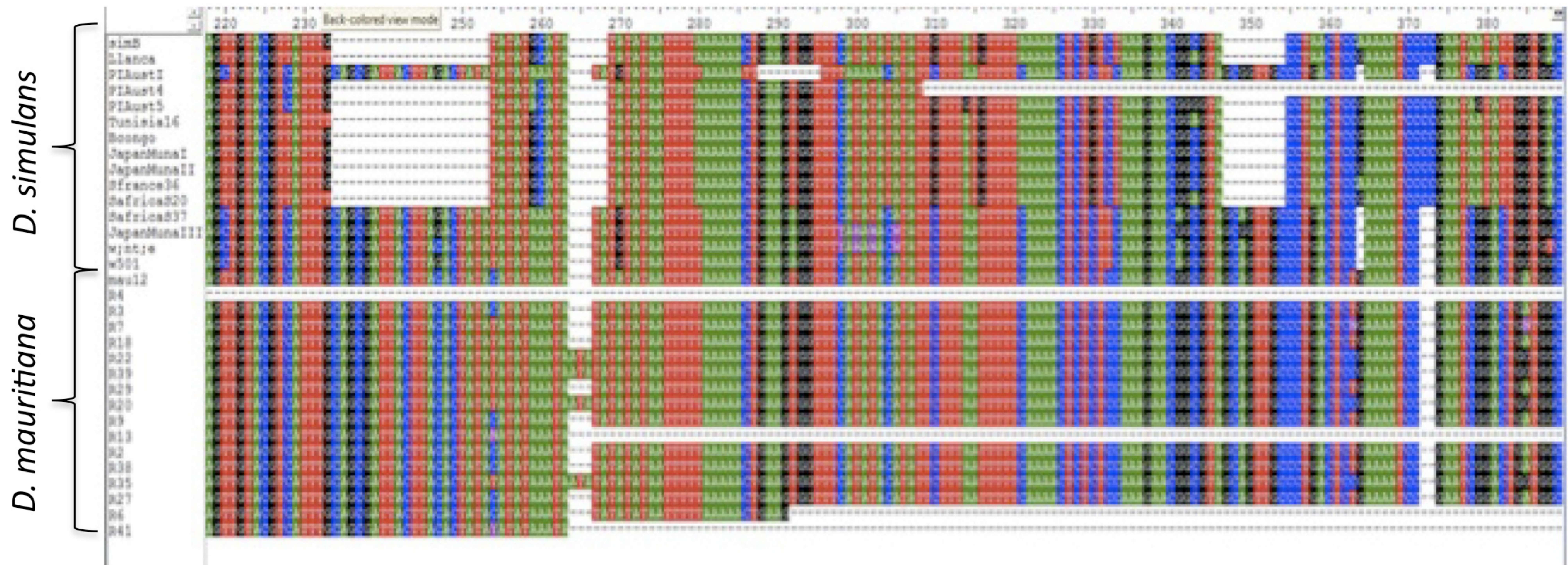

Supplement: Additional file 2 — Graphic scheme of the region where factor 1 is located. (A) Graphic scheme showing the 9 kb mapped region and the genes found within it (gene span and mRNA). Note that only seven of Taf1's 16 exons are contained in the region. The arrows show approximate location and relative size of indels found in the upstream region of gene agt. (B) alignment of a portion of the upstream region of gene agt for different populations of D. simulans and D. mauritiana. [file 1471-2148-10-385-S2.PDF]

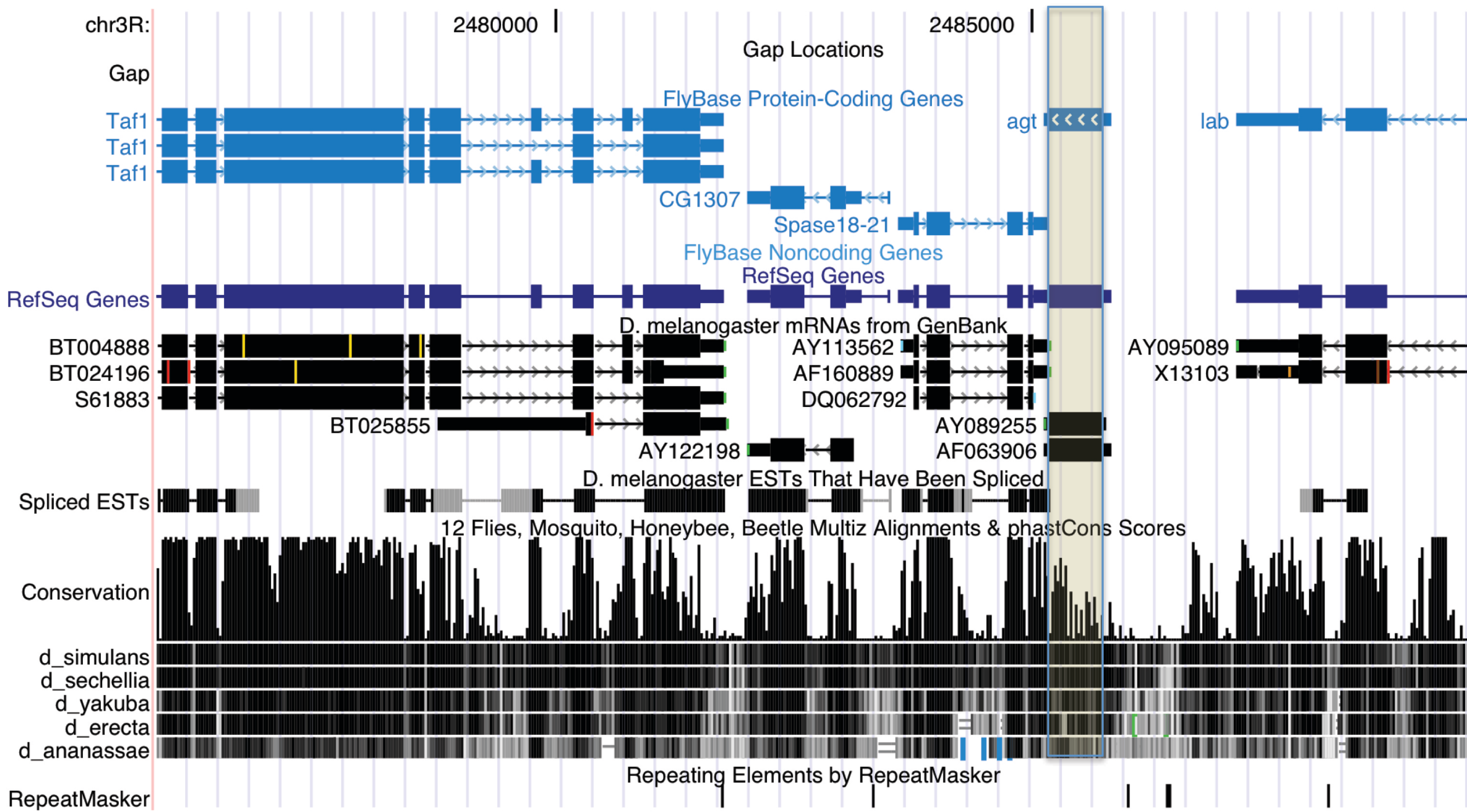

Supplement: Additional file 3 — Inter-species conservation across the mapped region. Graph from UCSC alignments showing the degree of conservation across species in D. melanogaster group and close species. The reference sequence represents the species D. melanogaster. A range of 20 kb is shown. Coding regions show much higher conservation than introns and intergenic regions. However, the coding region of gene agt shows low conservation across species (yellow stripe). [file 1471-2148-10-385-S3.PDF]
