# Supplementary material for: Fine-scale genetic mapping of a hybrid sterility factor between Drosophila simulans and D. mauritiana: the varied and elusive functions of "speciation genes"
Source: BMC Evol Biol. 2010 Dec 14;10:385. doi: 10.1186/1471-2148-10-385 (PMC3020225; doi:10.1186/1471-2148-10-385)
Supplement: Additional file 4 — List of genes showing misexpression in at least one of the non-fertile lines. [file 1471-2148-10-385-S4.PDF]

List of genes showing misexpression in at least one of the non-fertile lines

FBgn0000078
FBgn0000079
FBgn0000171
FBgn0000173
FBgn0000229
FBgn0000274
FBgn0000279
FBgn0000313
FBgn0000319
FBgn0000404
FBgn0000416
FBgn0000454
FBgn0000455
FBgn0000477
FBgn0000536
FBgn0000556
FBgn0000565
FBgn0000615
FBgn0001089
FBgn0001104
FBgn0001112
FBgn0001139
FBgn0001203
FBgn0001208
FBgn0001224
FBgn0001225
FBgn0001281
FBgn0001285
FBgn0002069
FBgn0002570
FBgn0002571
FBgn0002573
FBgn0002578
FBgn0002673
FBgn0002778
FBgn0002865
FBgn0002936
FBgn0002939
FBgn0003016
FBgn0003150
FBgn0003200
FBgn0003261
FBgn0003302
FBgn0003356
FBgn0003357
FBgn0003358
FBgn0003435

FBgn0003514
FBgn0003715
FBgn0003748
FBgn0003863
FBgn0003889
FBgn0003890
FBgn0003961
FBgn0003965
FBgn0004002
FBgn0004055
FBgn0004106
FBgn0004173
FBgn0004174
FBgn0004175
FBgn0004228
FBgn0004240
FBgn0004373
FBgn0004414
FBgn0004425
FBgn0004428
FBgn0004429
FBgn0004431
FBgn0004461
FBgn0004516
FBgn0004643
FBgn0004654
FBgn0005626
FBgn0005648
FBgn0005671
FBgn0005683
FBgn0010053
FBgn0010223
FBgn0010225
FBgn0010241
FBgn0010258
FBgn0010314
FBgn0010425
FBgn0010470
FBgn0010548
FBgn0010609
FBgn0010651
FBgn0010803
FBgn0011253
FBgn0011273
FBgn0011284
FBgn0011555
FBgn0011576
FBgn0011598
FBgn0011672

FBgn0011823
FBgn0011834
FBgn0012034
FBgn0012037
FBgn0012042
FBgn0013272
FBgn0013300
FBgn0013301
FBgn0013305
FBgn0013307
FBgn0013347
FBgn0013770
FBgn0013987
FBgn0014019
FBgn0014380
FBgn0014469
FBgn0014865
FBgn0015001
FBgn0015033
FBgn0015035
FBgn0015039
FBgn0015221
FBgn0015575
FBgn0015576
FBgn0015714
FBgn0015797
FBgn0015924
FBgn0016013
FBgn0016123
FBgn0016126
FBgn0016672
FBgn0016684
FBgn0016718
FBgn0019886
FBgn0019925
FBgn0019940
FBgn0019952
FBgn0020224
FBgn0020236
FBgn0020300
FBgn0020367
FBgn0020439
FBgn0020506
FBgn0020508
FBgn0020653
FBgn0020765
FBgn0020906
FBgn0020907
FBgn0020910

FBgn0021875
FBgn0022073
FBgn0022355
FBgn0023023
FBgn0023169
FBgn0023197
FBgn0023441
FBgn0023477
FBgn0023479
FBgn0023507
FBgn0023531
FBgn0023541
FBgn0024289
FBgn0024294
FBgn0024315
FBgn0024330
FBgn0024352
FBgn0024807
FBgn0024889
FBgn0024997
FBgn0025454
FBgn0025593
FBgn0025620
FBgn0025640
FBgn0025680
FBgn0025687
FBgn0025692
FBgn0025704
FBgn0025821
FBgn0025839
FBgn0026077
FBgn0026085
FBgn0026176
FBgn0026314
FBgn0026401
FBgn0026439
FBgn0026585
FBgn0026620
FBgn0026721
FBgn0027081
FBgn0027094
FBgn0027106
FBgn0027348
FBgn0027493
FBgn0027498
FBgn0027532
FBgn0027560
FBgn0027568
FBgn0027572

FBgn0027580
FBgn0027586
FBgn0027589
FBgn0027599
FBgn0027611
FBgn0027657
FBgn0027885
FBgn0027936
FBgn0027949
FBgn0028327
FBgn0028381
FBgn0028399
FBgn0028406
FBgn0028407
FBgn0028433
FBgn0028473
FBgn0028480
FBgn0028490
FBgn0028491
FBgn0028516
FBgn0028520
FBgn0028526
FBgn0028542
FBgn0028563
FBgn0028582
FBgn0028583
FBgn0028665
FBgn0028844
FBgn0028853
FBgn0028870
FBgn0028872
FBgn0028883
FBgn0028886
FBgn0028899
FBgn0028945
FBgn0028949
FBgn0028950
FBgn0028980
FBgn0028986
FBgn0028997
FBgn0029092
FBgn0029147
FBgn0029172
FBgn0029507
FBgn0029608
FBgn0029752
FBgn0029812
FBgn0029821
FBgn0029827

FBgn0029828
FBgn0029831
FBgn0029849
FBgn0029885
FBgn0029896
FBgn0029914
FBgn0029927
FBgn0029930
FBgn0029932
FBgn0029945
FBgn0029946
FBgn0029952
FBgn0029969
FBgn0029980
FBgn0029987
FBgn0029994
FBgn0030040
FBgn0030050
FBgn0030051
FBgn0030098
FBgn0030158
FBgn0030160
FBgn0030241
FBgn0030245
FBgn0030251
FBgn0030258
FBgn0030277
FBgn0030334
FBgn0030366
FBgn0030374
FBgn0030434
FBgn0030484
FBgn0030599
FBgn0030608
FBgn0030620
FBgn0030720
FBgn0030734
FBgn0030765
FBgn0030827
FBgn0030829
FBgn0030837
FBgn0030929
FBgn0030968
FBgn0030985
FBgn0030989
FBgn0030999
FBgn0031059
FBgn0031141
FBgn0031214

FBgn0031245
FBgn0031247
FBgn0031282
FBgn0031313
FBgn0031327
FBgn0031345
FBgn0031362
FBgn0031365
FBgn0031391
FBgn0031405
FBgn0031418
FBgn0031432
FBgn0031442
FBgn0031489
FBgn0031538
FBgn0031575
FBgn0031581
FBgn0031590
FBgn0031623
FBgn0031636
FBgn0031653
FBgn0031654
FBgn0031657
FBgn0031702
FBgn0031741
FBgn0031751
FBgn0031757
FBgn0031758
FBgn0031791
FBgn0031813
FBgn0031849
FBgn0031859
FBgn0031865
FBgn0031880
FBgn0031910
FBgn0031913
FBgn0031914
FBgn0031918
FBgn0031930
FBgn0031934
FBgn0031950
FBgn0031968
FBgn0031972
FBgn0031998
FBgn0032010
FBgn0032012
FBgn0032025
FBgn0032039
FBgn0032055

FBgn0032074
FBgn0032075
FBgn0032079
FBgn0032105
FBgn0032111
FBgn0032135
FBgn0032144
FBgn0032161
FBgn0032167
FBgn0032200
FBgn0032214
FBgn0032216
FBgn0032219
FBgn0032235
FBgn0032330
FBgn0032336
FBgn0032345
FBgn0032360
FBgn0032368
FBgn0032371
FBgn0032373
FBgn0032412
FBgn0032439
FBgn0032464
FBgn0032520
FBgn0032522
FBgn0032553
FBgn0032609
FBgn0032625
FBgn0032646
FBgn0032659
FBgn0032665
FBgn0032723
FBgn0032727
FBgn0032731
FBgn0032773
FBgn0032774
FBgn0032775
FBgn0032782
FBgn0032787
FBgn0032839
FBgn0032869
FBgn0032889
FBgn0032913
FBgn0032915
FBgn0032943
FBgn0032945
FBgn0032947
FBgn0032949

FBgn0032960
FBgn0032971
FBgn0032972
FBgn0033047
FBgn0033050
FBgn0033065
FBgn0033079
FBgn0033093
FBgn0033124
FBgn0033128
FBgn0033138
FBgn0033139
FBgn0033141
FBgn0033190
FBgn0033232
FBgn0033241
FBgn0033268
FBgn0033274
FBgn0033286
FBgn0033287
FBgn0033297
FBgn0033302
FBgn0033353
FBgn0033367
FBgn0033371
FBgn0033387
FBgn0033393
FBgn0033402
FBgn0033423
FBgn0033468
FBgn0033541
FBgn0033548
FBgn0033574
FBgn0033613
FBgn0033623
FBgn0033638
FBgn0033648
FBgn0033656
FBgn0033659
FBgn0033717
FBgn0033723
FBgn0033730
FBgn0033733
FBgn0033760
FBgn0033774
FBgn0033777
FBgn0033787
FBgn0033788
FBgn0033789

FBgn0033795
FBgn0033798
FBgn0033800
FBgn0033812
FBgn0033814
FBgn0033821
FBgn0033837
FBgn0033857
FBgn0033861
FBgn0033875
FBgn0033879
FBgn0033885
FBgn0033952
FBgn0033953
FBgn0033954
FBgn0033978
FBgn0033981
FBgn0033999
FBgn0034052
FBgn0034067
FBgn0034099
FBgn0034104
FBgn0034117
FBgn0034147
FBgn0034151
FBgn0034160
FBgn0034202
FBgn0034225
FBgn0034229
FBgn0034237
FBgn0034247
FBgn0034276
FBgn0034277
FBgn0034292
FBgn0034295
FBgn0034296
FBgn0034328
FBgn0034335
FBgn0034368
FBgn0034388
FBgn0034390
FBgn0034394
FBgn0034398
FBgn0034405
FBgn0034406
FBgn0034407
FBgn0034410
FBgn0034416
FBgn0034451

FBgn0034455
FBgn0034474
FBgn0034480
FBgn0034488
FBgn0034490
FBgn0034512
FBgn0034563
FBgn0034582
FBgn0034595
FBgn0034605
FBgn0034638
FBgn0034647
FBgn0034658
FBgn0034659
FBgn0034660
FBgn0034663
FBgn0034664
FBgn0034710
FBgn0034711
FBgn0034715
FBgn0034716
FBgn0034741
FBgn0034753
FBgn0034758
FBgn0034760
FBgn0034761
FBgn0034776
FBgn0034784
FBgn0034796
FBgn0034802
FBgn0034808
FBgn0034824
FBgn0034837
FBgn0034838
FBgn0034851
FBgn0034863
FBgn0034883
FBgn0034885
FBgn0034928
FBgn0034958
FBgn0034998
FBgn0035040
FBgn0035047
FBgn0035133
FBgn0035138
FBgn0035140
FBgn0035154
FBgn0035161
FBgn0035189

FBgn0035199
FBgn0035211
FBgn0035238
FBgn0035252
FBgn0035357
FBgn0035358
FBgn0035359
FBgn0035360
FBgn0035375
FBgn0035399
FBgn0035426
FBgn0035432
FBgn0035444
FBgn0035455
FBgn0035471
FBgn0035476
FBgn0035481
FBgn0035482
FBgn0035484
FBgn0035499
FBgn0035526
FBgn0035532
FBgn0035534
FBgn0035539
FBgn0035542
FBgn0035558
FBgn0035574
FBgn0035584
FBgn0035585
FBgn0035664
FBgn0035665
FBgn0035666
FBgn0035667
FBgn0035670
FBgn0035673
FBgn0035679
FBgn0035708
FBgn0035709
FBgn0035736
FBgn0035742
FBgn0035743
FBgn0035744
FBgn0035779
FBgn0035781
FBgn0035782
FBgn0035791
FBgn0035831
FBgn0035850
FBgn0035857

FBgn0035868
FBgn0035872
FBgn0035886
FBgn0035887
FBgn0035900
FBgn0035904
FBgn0035909
FBgn0035930
FBgn0035957
FBgn0035979
FBgn0035985
FBgn0036056
FBgn0036090
FBgn0036091
FBgn0036096
FBgn0036110
FBgn0036115
FBgn0036153
FBgn0036160
FBgn0036164
FBgn0036182
FBgn0036213
FBgn0036232
FBgn0036262
FBgn0036286
FBgn0036301
FBgn0036316
FBgn0036327
FBgn0036328
FBgn0036422
FBgn0036454
FBgn0036482
FBgn0036490
FBgn0036497
FBgn0036498
FBgn0036499
FBgn0036573
FBgn0036609
FBgn0036619
FBgn0036622
FBgn0036656
FBgn0036663
FBgn0036708
FBgn0036727
FBgn0036738
FBgn0036756
FBgn0036766
FBgn0036767
FBgn0036806

FBgn0036815
FBgn0036816
FBgn0036825
FBgn0036833
FBgn0036857
FBgn0036861
FBgn0036927
FBgn0036928
FBgn0037007
FBgn0037046
FBgn0037063
FBgn0037070
FBgn0037071
FBgn0037146
FBgn0037149
FBgn0037166
FBgn0037168
FBgn0037176
FBgn0037202
FBgn0037249
FBgn0037292
FBgn0037323
FBgn0037342
FBgn0037354
FBgn0037358
FBgn0037375
FBgn0037387
FBgn0037389
FBgn0037440
FBgn0037460
FBgn0037472
FBgn0037486
FBgn0037504
FBgn0037506
FBgn0037547
FBgn0037553
FBgn0037560
FBgn0037572
FBgn0037607
FBgn0037613
FBgn0037643
FBgn0037653
FBgn0037676
FBgn0037678
FBgn0037683
FBgn0037744
FBgn0037765
FBgn0037783
FBgn0037788

FBgn0037818
FBgn0037875
FBgn0037884
FBgn0037888
FBgn0037911
FBgn0037924
FBgn0037936
FBgn0037962
FBgn0037969
FBgn0037973
FBgn0037974
FBgn0037975
FBgn0037994
FBgn0038038
FBgn0038043
FBgn0038052
FBgn0038072
FBgn0038078
FBgn0038100
FBgn0038136
FBgn0038179
FBgn0038195
FBgn0038200
FBgn0038209
FBgn0038293
FBgn0038307
FBgn0038347
FBgn0038353
FBgn0038368
FBgn0038395
FBgn0038428
FBgn0038449
FBgn0038450
FBgn0038465
FBgn0038481
FBgn0038482
FBgn0038484
FBgn0038490
FBgn0038515
FBgn0038516
FBgn0038525
FBgn0038535
FBgn0038581
FBgn0038631
FBgn0038643
FBgn0038649
FBgn0038652
FBgn0038681
FBgn0038685

FBgn0038718
FBgn0038739
FBgn0038742
FBgn0038857
FBgn0038878
FBgn0038903
FBgn0038914
FBgn0038923
FBgn0038924
FBgn0038944
FBgn0038966
FBgn0038974
FBgn0038979
FBgn0039073
FBgn0039094
FBgn0039104
FBgn0039114
FBgn0039141
FBgn0039219
FBgn0039241
FBgn0039298
FBgn0039307
FBgn0039310
FBgn0039313
FBgn0039315
FBgn0039324
FBgn0039326
FBgn0039330
FBgn0039342
FBgn0039348
FBgn0039349
FBgn0039359
FBgn0039369
FBgn0039373
FBgn0039395
FBgn0039471
FBgn0039472
FBgn0039474
FBgn0039475
FBgn0039476
FBgn0039486
FBgn0039498
FBgn0039504
FBgn0039518
FBgn0039593
FBgn0039622
FBgn0039678
FBgn0039686
FBgn0039725

FBgn0039738
FBgn0039761
FBgn0039776
FBgn0039777
FBgn0039797
FBgn0039801
FBgn0039819
FBgn0039858
FBgn0039905
FBgn0039927
FBgn0039965
FBgn0040064
FBgn0040074
FBgn0040256
FBgn0040259
FBgn0040260
FBgn0040297
FBgn0040299
FBgn0040308
FBgn0040336
FBgn0040339
FBgn0040349
FBgn0040383
FBgn0040493
FBgn0040519
FBgn0040528
FBgn0040705
FBgn0040723
FBgn0040732
FBgn0040747
FBgn0040777
FBgn0040813
FBgn0040814
FBgn0040816
FBgn0040871
FBgn0040958
FBgn0040959
FBgn0040994
FBgn0041102
FBgn0041105
FBgn0041180
FBgn0041182
FBgn0041194
FBgn0041579
FBgn0041630
FBgn0042092
FBgn0042133
FBgn0042146
FBgn0042630

FBgn0043470
FBgn0043471
FBgn0043575
FBgn0043792
FBgn0044030
FBgn0045770
FBgn0045866
FBgn0046294
FBgn0046297
FBgn0046302
FBgn0046763
FBgn0050004
FBgn0050011
FBgn0050046
FBgn0050072
FBgn0050075
FBgn0050154
FBgn0050360
FBgn0050361
FBgn0050376
FBgn0050428
FBgn0050447
FBgn0050476
FBgn0050486
FBgn0051025
FBgn0051029
FBgn0051063
FBgn0051075
FBgn0051092
FBgn0051099
FBgn0051104
FBgn0051106
FBgn0051198
FBgn0051205
FBgn0051233
FBgn0051265
FBgn0051266
FBgn0051288
FBgn0051300
FBgn0051315
FBgn0051343
FBgn0051358
FBgn0051363
FBgn0051469
FBgn0051562
FBgn0051601
FBgn0051639
FBgn0051674
FBgn0051709

FBgn0051740
FBgn0051751
FBgn0051769
FBgn0051788
FBgn0051797
FBgn0051872
FBgn0051948
FBgn0051949
FBgn0051960
FBgn0051974
FBgn0052030
FBgn0052068
FBgn0052086
FBgn0052087
FBgn0052100
FBgn0052203
FBgn0052240
FBgn0052352
FBgn0052380
FBgn0052425
FBgn0052432
FBgn0052483
FBgn0052572
FBgn0052675
FBgn0052687
FBgn0052700
FBgn0052736
FBgn0052808
FBgn0052986
FBgn0053054
FBgn0053080
FBgn0053100
FBgn0053109
FBgn0053198
FBgn0053209
FBgn0053258
FBgn0053281
FBgn0053340
FBgn0053514
FBgn0053515
FBgn0053542
FBgn0053715
FBgn0054035
FBgn0063449
FBgn0063493
FBgn0063494
FBgn0063496
FBgn0063499
FBgn0063667

FBgn0064237
FBgn0065032
FBgn0083919
