# Supplementary material for: Fine-scale genetic mapping of a hybrid sterility factor between Drosophila simulans and D. mauritiana: the varied and elusive functions of "speciation genes"
Source: BMC Evol Biol. 2010 Dec 14;10:385. doi: 10.1186/1471-2148-10-385 (PMC3020225; doi:10.1186/1471-2148-10-385)
Supplement: Additional file 5 — List of genes with misexpression congruent in all four non-fertile lines. Negative values mean that genes were down regulated in the non-fertile lines in relation to the fertile one and positive values mean up regulation in the non-fertile lines. The 5 down regulated genes contained within the introgressed segment are shown in red. [file 1471-2148-10-385-S5.PDF]

List of genes with misexpression congruent in all four non-fertile lines

	Fertile vs NF1	Fertile vs NF2	Fertile vs NF3	Fertile vs NF4
FBgn0000078	-1	-1	-1	-1
FBgn0000079	-1	-1	-1	-1
FBgn0000404	1	1	1	1
FBgn0000615	1	1	1	1
FBgn0001089	-1	-1	-1	-1
FBgn0001225	1	1	1	1
FBgn0001285	-1	-1	-1	-1
FBgn0002570	-1	-1	-1	-1
FBgn0002571	-1	-1	-1	-1
FBgn0002578	-1	-1	-1	-1
FBgn0002673	1	1	1	1
FBgn0002939	-1	-1	-1	-1
FBgn0003356	-1	-1	-1	-1
FBgn0003358	-1	-1	-1	-1
FBgn0003863	-1	-1	-1	-1
FBgn0004173	-1	-1	-1	-1
FBgn0004174	-1	-1	-1	-1
FBgn0005683	1	1	1	1
FBgn0010053	-1	-1	-1	-1
FBgn0010225	-1	-1	-1	-1
FBgn0010425	-1	-1	-1	-1
FBgn0010548	-1	-1	-1	-1
FBgn0010651	-1	-1	-1	-1
FBgn0011555	-1	-1	-1	-1
FBgn0011598	1	1	1	1
FBgn0014469	-1	-1	-1	-1
FBgn0015039	-1	-1	-1	-1
FBgn0015575	-1	-1	-1	-1
FBgn0015576	-1	-1	-1	-1
FBgn0015924	-1	-1	-1	-1
FBgn0020367	-1	-1	-1	-1
FBgn0020906	-1	-1	-1	-1
FBgn0023197	-1	-1	-1	-1
FBgn0023541	-1	-1	-1	-1
FBgn0025692	-1	-1	-1	-1
FBgn0026085	1	1	1	1
FBgn0027081	-1	-1	-1	-1
FBgn0027532	-1	-1	-1	-1
FBgn0027611	-1	-1	-1	-1
FBgn0028491	-1	-1	-1	-1
FBgn0028844	1	1	1	1
FBgn0028853	-1	-1	-1	-1
FBgn0029147	-1	-1	-1	-1
FBgn0029827	-1	-1	-1	-1
FBgn0029930	-1	1	1	1
FBgn0029932	-1	-1	-1	-1

FBgn0030040	-1	-1	-1	-1
FBgn0030241	1	1	1	1
FBgn0030334	-1	-1	-1	-1
FBgn0030484	-1	-1	-1	-1
FBgn0030765	1	1	1	1
FBgn0030837	-1	-1	-1	-1
FBgn0031313	-1	-1	-1	-1
FBgn0031418	-1	-1	-1	-1
FBgn0031636	-1	-1	-1	-1
FBgn0031654	-1	-1	-1	-1
FBgn0031741	-1	-1	-1	-1
FBgn0031813	-1	-1	-1	-1
FBgn0032235	-1	-1	-1	-1
FBgn0032439	1	1	1	1
FBgn0032464	-1	-1	-1	-1
FBgn0032665	-1	-1	-1	-1
FBgn0033047	-1	-1	-1	-1
FBgn0033124	-1	-1	-1	-1
FBgn0033138	-1	-1	-1	-1
FBgn0033297	-1	-1	-1	-1
FBgn0033367	-1	-1	-1	-1
FBgn0033423	-1	-1	-1	-1
FBgn0033541	-1	-1	-1	-1
FBgn0033723	-1	-1	-1	-1
FBgn0033733	-1	-1	-1	-1
FBgn0033774	-1	-1	-1	-1
FBgn0033789	-1	-1	-1	-1
FBgn0033821	-1	-1	-1	-1
FBgn0033861	1	1	1	1
FBgn0033954	-1	-1	-1	-1
FBgn0033978	-1	-1	-1	-1
FBgn0034202	-1	-1	-1	-1
FBgn0034225	-1	-1	-1	-1
FBgn0034247	-1	-1	-1	-1
FBgn0034406	-1	-1	-1	-1
FBgn0034480	-1	-1	-1	-1
FBgn0034582	-1	-1	-1	-1
FBgn0034663	-1	-1	-1	-1
FBgn0034998	1	1	1	1
FBgn0035133	1	1	1	1
FBgn0035360	-1	-1	-1	-1
FBgn0035399	1	1	1	1
FBgn0035471	-1	-1	-1	-1
FBgn0035481	1	1	1	1
FBgn0035585	1	1	1	1
FBgn0035664	-1	-1	-1	-1
FBgn0035665	-1	-1	-1	-1
FBgn0035666	-1	-1	-1	-1
FBgn0035670	-1	-1	-1	-1

FBgn0035743	-1	-1	-1	-1
FBgn0035857	1	1	1	1
FBgn0035868	-1	-1	-1	-1
FBgn0036091	1	1	1	1
FBgn0036622	-1	-1	-1	-1
FBgn0036738	-1	-1	-1	-1
FBgn0036756	-1	-1	-1	-1
FBgn0036833	-1	-1	-1	-1
FBgn0037202	1	1	1	1
FBgn0037292	1	1	1	1
FBgn0037358	-1	-1	-1	-1
FBgn0037387	-1	-1	-1	-1
FBgn0037389	1	1	1	1
FBgn0037440	-1	-1	-1	-1
FBgn0037504	-1	-1	-1	-1
FBgn0037506	-1	-1	-1	-1
FBgn0037765	-1	-1	-1	-1
FBgn0037788	-1	-1	-1	-1
FBgn0037936	-1	-1	-1	-1
FBgn0038038	-1	-1	-1	-1
FBgn0038209	1	1	1	1
FBgn0038347	-1	-1	-1	-1
FBgn0038368	1	1	1	1
FBgn0038465	-1	-1	-1	-1
FBgn0038643	1	1	1	1
FBgn0038649	1	1	1	1
FBgn0038652	-1	-1	-1	-1
FBgn0038718	-1	-1	-1	-1
FBgn0038878	-1	-1	-1	-1
FBgn0039094	-1	-1	-1	-1
FBgn0039315	-1	-1	-1	-1
FBgn0039326	-1	-1	-1	-1
FBgn0039330	-1	-1	-1	-1
FBgn0039342	-1	-1	-1	-1
FBgn0039348	1	1	1	1
FBgn0039471	-1	-1	-1	-1
FBgn0039472	-1	-1	-1	-1
FBgn0039474	-1	-1	-1	-1
FBgn0039475	-1	-1	-1	-1
FBgn0039476	-1	-1	-1	-1
FBgn0039498	1	1	1	1
FBgn0039686	1	1	1	1
FBgn0039777	-1	-1	-1	-1
FBgn0039905	-1	-1	-1	-1
FBgn0040259	-1	-1	-1	-1
FBgn0040871	1	1	1	1
FBgn0041194	-1	-1	-1	-1
FBgn0043470	-1	-1	-1	-1
FBgn0043471	-1	-1	-1	-1

FBgn0046302	-1	-1	-1	-1
FBgn0050072	1	1	1	1
FBgn0050360	-1	-1	-1	-1
FBgn0051233	-1	-1	-1	-1
FBgn0051288	-1	-1	-1	-1
FBgn0051562	-1	-1	-1	-1
FBgn0051601	1	1	1	1
FBgn0051639	-1	-1	-1	-1
FBgn0051674	-1	-1	-1	-1
FBgn0052483	-1	-1	-1	-1
FBgn0052700	-1	-1	-1	-1
FBgn0052986	1	1	1	1
FBgn0064237	-1	-1	-1	-1
