# Supplementary material for: Fine-scale genetic mapping of a hybrid sterility factor between Drosophila simulans and D. mauritiana: the varied and elusive functions of "speciation genes"
Source: BMC Evol Biol. 2010 Dec 14;10:385. doi: 10.1186/1471-2148-10-385 (PMC3020225; doi:10.1186/1471-2148-10-385)
Supplement: Additional file 6 — List of molecular markers used for mapping factor 1. [file 1471-2148-10-385-S6.PDF]

List of molecular markers used for mapping *factor 1*

**ASO marker**	Cytological position	PCR Primers F/R	ASO probes	Wash temperature sim/mau
Rga	83B	GATGGTGAGTGCTGGAACACTTC/<br>GTTAATCAGATATTCGGGTGGCAC	S: CAACTTTCCTGGTAC<br>M: CAACCTTCCTGTTAC	37°C/45°C
CG1077	83D5	GACAGCACCAAGATGAAGCA/<br>TTGTGTGATTGATGCCGTTT	S: GTCATCGATTTAAAGCCTA<br>M: TAGGCTTAAAATCAATGAC	55°C/60°C
CG10277	83E5	ACGGTTGTGATAGCCTGGAC/<br>CACTGGCAATTTCTTCAGCA	S: GCTCTGTAATTGAATATG<br>M: CATATTCAACTACAGAGC	55°C/65°C
CG15179	84A1	ATGAACCGCATCGACCTTAC/<br>TCCGAATATGAACTCGCACA	S: CATGAACTACAGGTTTA<br>M: TAAACCTGTAATCCATG	55°C/65°C
CG1307	84A1	GCCAGCACAGTCTTGGAGTT/<br>TTTTCGTGGGCTACCGATAC	S: AGTTCAGAATACCAAGT M:<br>ACTTGGTACTCTGAACT	55°C/55°C
lab	84A1	TGATCAATAAATCCCCGAAAA/<br>GCCACCACAACTCGTCTGTA	S: TAACAATTTGCAATCGG<br>M: CCGACTGCAAATTGTTA	55°C/55°C
Ccp84ad	84A3	TTTCAGTTCATCGCCCTCTT/<br>CGGCGTAGGAGGTGTAGGTA	S: AAGACCGTCGCCGCT<br>M: AGCTGCGACGGTCTT	60°C/60°C
Dfd	84A5	AAATCGGCTCGAATGGAAC/<br>AGTGCGGATGTTGCTATGGT	S: GATAGTGCCCAAGTAAAAT<br>M: ATTTTTCTTGGACACTATC	55°C/57°C
pb	84A5	GGACTTGTGCGAAAGTCGTT/<br>AGAACTGCGAGGGCTACAAC	S: AGCTCGGACTTCAACTTT<br>M: AAAGTTAAAGTCCGAGCT	60°C/60°C
Antp	84B	ACGGACGTTGGAGTTCCCGA/<br>ACATGCCCATGTTGTGATGG	S: CACCTACTTCAACTT<br>M: AAGTTAAAGTAAGTG	35°C/35°C
**PCR success/ failure**		Forward sim/mau	Reverse	Annealing Temp
DM9300	84A	ACGAGGATGGGCGTGCATGC/<br>ACGAGGGAGGGCGTGTGTGC	GTCGCCTTTATTGCAGTGCT	58°C
DM14869	84A	CACATCATTCGACGCCTTC/<br>CACATCATTCGACGCCTTT	TGCACATCAGCTACGAGTCC	52°C
DM18764	84A	ATTGGCATTTCCCAAGCTTC/<br>ACTGGCATCCCCAAGCTCCC	CTCCTTGCGGTCGATAACCT	55°C
DM15810	84A	GCCAAGTTGGACATAGATACG/<br>CCAAGTTAAATTTGTGCCTCAAG	CCCTGATAATGGCGAACAGT	52°C
DM36680	84A	AAAACAAGCCCCAGGATTACC/<br>AAAACAAGCCCCAGGATTACG	CACAGTGAAGCAGGAGTTGC	58°C
**PCR fragment length**		Forward	Reverse	Annealing Temp
Int14000	84A	GCAGTGGTACAATTTGGCAAT	CAGTTGAGAAACAAAAAGCAGTG	55°C
**SNP position on sequence**		mau	sim	
pos3472	84A	GAAGCAGTG**T**GCCGACTTCA	GAAGCAGTG**C**GCCGACTTCA	
pos5279	84A	GAAGAAACG**C**AGAGTGGGCG	GAAGAAACG**T**AGAGTGGGCG	
pos14460	84A	CTGTTGCA::::GATCAATA	CTGTTGCAAAAAGATCAATA	
